# Supportive Care Needs Assessment for Cancer Survivors at a Comprehensive Cancer Center in the Middle East: Mending the Gap

**DOI:** 10.3390/cancers14041002

**Published:** 2022-02-16

**Authors:** Amal Al-Omari, Nedal Al-Rawashdeh, Rana Damsees, Khawlah Ammar, Ibrahim Alananzeh, Bayan Inserat, Dalia Al-Rimawi, Shrouq Tbayshat, Hazim Ababneh, Haneen Alishreim, Hashem Abu Serhan, Faisal Al-Noaaimi, Hikmat Abdel-Razeq

**Affiliations:** 1 The Office of Scientific Affairs and Research, King Hussein Cancer Center, Amman 11941, Jordan; na.08085@khcc.jo (N.A.-R.); ra.13579@khcc.jo (R.D.); ka.11148@khcc.jo (K.A.); bi.14129@khcc.jo (B.I.); drimawi@khcc.jo (D.A.-R.); hazem.ababneh@gmail.com (H.A.); haneenghalib95@gmail.com (H.A.); hashemabusarhan@yahoo.com (H.A.S.); faisal.n3eamy@gmail.com (F.A.-N.); 2 School of Nursing, Faculty of Science, Medicine and Health, University of Wollongong, Northfields Avenue, Wollongong, NSW 2522, Australia; ibrahima@uow.edu.au; 3 Department of Internal Medicine, King Hussein Cancer Center, Amman 11941, Jordan; st.13501@khcc.jo (S.T.); habdelrazeq@khcc.jo (H.A.-R.)

**Keywords:** cancer survivors, supportive care, needs assessment, quality of life, Arab population

## Abstract

**Simple Summary:**

There are knowledge gaps regarding supportive care needs of cancer survivors in Jordan and the Arab region. Assessing unmet needs is crucial to achieving quality cancer care and patient satisfaction. In this study, we aimed to identify gaps in supportive care needs among adult cancer survivors seen at King Hussein Cancer Center in Amman, Jordan, explore predictors of unmet needs and assess the relationship between unmet supportive care needs and quality of life of adult cancer survivors. We confirmed the presence of several unmet needs in this population of cancer survivors that were evident in many domains. Late-stage diagnosis and quality of life as reported by study participants provided additional and independent information for unmet needs in several domains. Overall, this needs assessment identified problem areas for targeting interventions across the Jordanian cancer survivor population and understanding these findings highlights opportunities for intervention to address gaps in care.

**Abstract:**

Background: Cancer survivors are often underprepared for what to expect post-treatment, and there are knowledge gaps regarding cancer survivors’ supportive care needs in Jordan and neighboring Arab countries. This study aimed to identify gaps in supportive care needs among adult cancer survivors seen at King Hussein Cancer Center in Amman, Jordan, and explore predictors of unmet needs. Methods: This was an observational cross-sectional study using a modified version of the Supportive Care Needs Survey 34 item short form (SCNS-SF34). Results: Two hundred and forty adult cancer survivors completed the study questionnaire. The assessed needs were highest in the financial domain, including covering living expenses, managing cancer treatment adverse effects and co-morbidities. The least prevalent reported needs were in sexuality and reproductive consultations. Late-stage diagnosis was independently associated with higher physical, psychological, health system/information, financial and overall need scores, with *p*-values of 0.032, 0.027, 0.052, 0.002 and 0.024, respectively. The overall quality of life score was independently and inversely associated with physical, psychological, health system/information, financial and overall need domains, with *p*-values of 0.015, <0.0001, 0.015, 0.004 and 0.0003, respectively. Conclusions: This needs assessment identified problem areas for targeting interventions across the Jordanian cancer survivor population, and understanding these findings highlights opportunities for intervention to address gaps in care.

## 1. Introduction

Cancer is a condition that causes cells to divide uncontrollably, resulting in tumor growth and immune system dysfunction. It is one of the main leading causes of death globally, and its incidence has increased in recent years due to a variety of factors, including population aging, tobacco use, exposure to radiation, adopting a more sedentary lifestyle and genetic predisposition [1,2,3,4,5,6]. In 2020, it is estimated that 19.3 million new cancer cases were diagnosed worldwide, with over 10.0 million cancer deaths. Despite the fact that female breast cancer is currently the most commonly diagnosed cancer, it only accounted for 6.9% of cancer fatalities in 2021. Lung cancer, with a predicted 1.8 million deaths, remained the top cause of cancer death, followed by colorectal, liver and stomach cancers [7]. The current advancement in cancer treatments and the evolving landscape of clinical trials increased the number of adults surviving cancer and improved their life expectancy [8,9,10].

Despite many challenges facing cancer treatment, screening, palliative care and cancer research at the national level in Jordan, survival rates at King Hussein Cancer Center (KHCC), the only comprehensive cancer center in the country, are similar to those observed in more developed countries [11]. Many cancer patients may experience varying degrees of long-term physical, social, financial, psychological and existential distress, complicating their survivorship with significant challenges [12,13]. Although some cancer-related concerns tend to decrease with time, some symptoms such as fatigue, pain and sleep difficulty persist, with some cancer survivors experiencing physical and/or psychological symptoms more than 10 years after treatment completion [14].

Supportive care is defined as providing basic services that satisfy the psychological, spiritual, physical, informational and social needs of cancer patients throughout the continuum of care. These supportive care aspects can help with functional and emotional adjustment, symptoms and quality of life among cancer patients and improve cancer survival [15,16,17]. Cancer survivors are often under-prepared for what to expect post-treatment, and the end of treatment is usually a vulnerable time for patients as they transition away from the support of their healthcare providers. Needs assessment is a direct measure of the gap between patients’ experience and their expectations and directly measures the magnitude of patients’ desire for help in dealing with the unmet needs.

The unmet healthcare needs of cancer survivors were previously addressed in several studies from North America, Europe, Japan, Australia and Asia, which reported that cancer patients have unmet psychological needs, in addition to the need for help with medical issues, information on cancer and late treatment effects. In a study conducted in Ireland, adult and childhood cancer patients have reported more psychosocial needs and socioeconomic concerns [18,19,20,21,22]. Similar results have been reported in the Asia-Pacific region [23]. In the United States, adult cancer survivors have reported a wide range of healthcare needs, including psychological needs, health systems/information, physical/daily living, patient care/support and sexuality. Younger female cancer patients reported a greater need than male cancer patients [24].

There are knowledge gaps regarding the needs of cancer survivors in Jordan and neighboring Arab countries, with very scarce literature addressing the topic. Very few studies conducted and highlighted few needs, such as physical and psychological, focusing on cancer patients in the first place as well as cancer survivors. In two studies conducted by Alananzeh et al., one was a review article of the Supportive Care Needs of Arab People affected by any type of cancer, and the other was an original research article aimed at exploring the unmet supportive care needs of both Arab-Australian and Arab-Jordanian cancer survivors [25,26].

In their review article, Alananzeh et al. identified three papers on Arab cancer patients’ needs, identifying the areas of information, symptom management, dependency and communication with healthcare providers and healthcare system navigation, two of which were conducted on Arab immigrants and one old paper that compared American and Egyptian cancer patients’ attitudes and unmet needs [27,28,29]. The original research was conducted in Jordan and Australia and was the only study that explored unmet supportive care needs among Arab cancer survivors. It was conducted on a very small sample size consisting of 77 Arab-Jordanian and 66 Arab-Australian cancer survivors, and found that Arab-Australians had a higher overall score for unmet needs compared to Arab-Jordanians. This study also revealed that physical and information/support needs were the most prevalent among Arab-Jordanians, unlike studies from the US, Europe, Japan, Australia and Asia, where unmet needs were mostly psychosocial and concerned with socioeconomic.

In the past, the effectiveness of cancer care has been assessed using biomedical endpoints, such as tumor response, survival and length of remission; the assessment of the quality of cancer patients’ survival has become more important. A decrease in quality of life among cancer patients has been shown to affect supportive care needs and vice versa. Several studies have found that a lower QoL is associated with more unmet needs. Giulian et al. found that a lower QoL was associated with higher levels of unmet needs across the five domains of the supportive care needs [30], Zhu et al. found that a lower QoL was associated with higher levels of unmet needs in the physical domain of the supportive care needs [31] and Molassiotis et al. revealed that a lower QoL was associated with greater needs in all five domains of the Cancer Survivors’ Unmet Needs measure [23]. In the present study, we investigated the direction of this association.

Assessing the gaps or unmet needs is crucial to achieving quality cancer care and patient satisfaction; therefore, in this study we aimed to identify gaps in supportive care needs among adult cancer survivors seen at KHCC, explore predictors of unmet needs and assess the relationship between unmet supportive care needs and the quality of life of adult cancer survivors. This study will help draw healthcare providers’ attention to the most common unmet needs among cancer survivors and understand areas in which they would like to receive help or assistance. Therefore, addressing these deficiencies is crucial to achieving optimal care and satisfaction.

## 2. Materials and Methods

### 2.1. Design

This was an observational cross-sectional study conducted between October 2020 and February 2021 at King Hussein Cancer Center (KHCC).

### 2.2. Study Setting

KHCC is a comprehensive cancer center in Amman, the capital city of Jordan, which treats more than 5000 new adult and pediatric patients annually from Jordan and other countries in the Middle East. Around two thirds of patients treated at KHCC are Jordanians, and the rest come from neighboring Arab countries, e.g., Palestinian territories, Iraq, Syria, Yemen, Libya and Sudan, among others. KHCC is the only specialized tertiary hospital that provides all treatment modalities and services for cancer care and adopts a multidisciplinary team management approach for case follow-up, including supportive psychosocial, nutrition, patient and family education and palliative care services.

### 2.3. Participants

We recruited a consecutive sample of adult cancer patients seen at KHCC. Patients were identified and recruited based on their follow-up appointments with the survivorship clinic. At KHCC, patients are referred to the survivorship clinic 1–2 years after completing their treatment and being deemed cancer-free; therefore, survivors were eligible for study participation if they were cancer-free and off treatment for not less than one year. Due to the COVID-19 pandemic, patients were invited to participate and consented via phone as per the KHCC internal distribution to minimize direct contact through applying virtual clinics as well the Institutional Review Board (IRB) decision to avoid direct contact with research subjects; the study questionnaire was then completed through a phone-based interview scheduled at a convenient time. Interview with patients starts by explaining the study using an IRB approved script and consent were taken verbally, then sociodemographic information, clinical information and financial consequences of cancer diagnosis were captured, followed by the 45 Supportive Care Needs questions, then the overall quality of life and the interview ends with open-ended questions to identify other needs and barriers to survivorship care. The participants were encouraged to sit in a relaxing place and to take breaks from the interview if they felt fatigued. The study received ethical approval from King Hussein Cancer Center’s Institutional Review Board under protocol number 20 KHCC 173F.

### 2.4. Survey Questionnaire

The survey instrument included questions on sociodemographics (e.g., age, gender, education, marital status, living status, income, place of residency), clinical information (e.g., age at diagnosis, date of diagnosis, cancer type, stage at diagnosis, other comorbidities) and financial consequences of cancer diagnosis (e.g., quitting jobs, change to part-time job, unable to cover the cost of treatment).

We basically used the validated Supportive Care Needs Survey 34 item short form (SCNS-SF34) in this study. The survey was designed to assess survivors’ needs across five domains: psychological, health system and information, physical and daily living, patient care and support and sexuality. Eleven additional items were added to the questionnaire to capture the needs of the KHCC patients. Six items were taken from the long-form SCNS-LF59, and the remaining five were recommended by the study team following the same Likert scale format used in the previous questions to cover fertility and financial needs based on experiences with cancer patients treated at KHCC.

Following the format of the SCNS-SF34, participants were asked to describe the level of help needed during the previous six months for each item in the questionnaire using a 5-point Likert scale (1—not applicable/not a problem, 2—satisfied, 3—low need, 4—moderate need, 5—high need). Values of 1 and 2 were rated as (No Need) and 3, 4 and 5 as (Some Need). Participants were also asked to rate their overall quality of life using the global health status/QoL, which are 7-point questions from 1 (very bad) to 7 (excellent), and to mention top other needs that were not covered in the questionnaire. The use of this simple tool provided us the opportunity to identify the quality of life and correlate the score with the healthcare needs among cancer survivors [32,33].

The original short-form (SCNS-SF34) as well as the questions taken from the long-form version (SCNS-LF59) were used in this study with no changes; both forms are validated tools that have been used widely in previous studies [34,35,36,37]. However, validity and reliability testing were conducted since we added five questions to the questionnaire. The validation process was performed by experts in the field, as well as all study team members, who reviewed the questionnaire thoroughly, and then changes were applied and were limited to those five added questions. After that, we piloted to test for reliability on 26 participants by calculating Cronbach’s α, and it was 0.958. To confirm the stability of the used study tool, Cronbach’s α was performed on the whole study sample, and it was 0.932 *n* = 240 in addition to the different domains, Physical 0.826, Psychological 0.860, Sexual 0.792, Patient care 0.792, Health system 0.905 and Financial 0.805.

### 2.5. Statistical Analysis

Descriptive statistics were used to characterize the distribution of sociodemographic characteristics, clinical information and unmet care needs of the population.

Raw need scores were standardized, taking the number of items in each domain into account. If m is the number of questions in the domain and k is the maximum value for each item (in this study k = 5), the standardized score for each domain is obtained by calculating (total raw score − m) × 100/[m × (k − 1)], so the score range for each domain will be from 0 to 100. Therefore, the higher the score on the domain, the higher the perceived need is for support in that domain [34,38]. Linear transformation was also used to standardize the overall quality of life (QoL) raw score, so that scores range from 0 to 100 using the following formula; score = (raw score − 1)/range × 100, where the range is the difference between the maximum possible value of raw score and the minimum possible value [39].

One Way ANOVA was used to compare means between different groups, followed by Bonferroni post hoc-test. Spearman’s correlation was used to test the association between overall quality of life score and mean need domain scores. A multivariate linear regression model was used to examine predictors of supportive care needs in each domain. Other needs were addressed using open-ended questions and were analyzed using content and thematic analysis to summarize the top unmet needs from a participant’s perspective. Missing data in this study were completely at random, therefore, they were ignored in the analysis. Statistical analyses were performed using SPSS for Windows, version 26.0 (SPSS Inc., Chicago, IL, USA), and all *p*-values are two-sided; *p*-value ≤ 0.05 is considered significant. The Bonferroni correction was applied after ANOVA test on variables with more than two levels, Age and Time, since a Diagnosis *p*-value ≤ 0.017 is considered significant, and Martial status and cancer type *p*-value ≤ 0.0083 is considered significant.

## 3. Results

### 3.1. Study Sample

A total of 347 participants were approached to be enrolled in this study, 52 did not pick their phones and 55 refused to participate. However, 240 questionnaires were completed by adult cancer survivors via phone. Participants were distributed across all Jordan governorates but the majority (160, 66.7%) were residing in the capital city Amman followed by Zarqa (29, 12.1%) and Irbid (24, 10%).

Mean age of participants (±SD) was 53.9 ± 12.4, median (range) = 53 (22–80) years. Around 75.5% were above the age of 45. Most of the cancer survivors in the study were females (69.6%). The majority of participants were married (78.3%), and nearly two-thirds reported an unemployment work status at the time of the survey (61.3%). The most prevalent cancer diagnosis was breast cancer (36.3%) followed by hematological malignancies (30.0%) and colorectal/gastrointestinal cancers (24.6%), and the majority reported an early-stage diagnosis (75.7%), Table 1. The mean overall quality of life (QoL) score was 68.6 ± 22.8. There was a significant difference in the overall QoL score reported by study participants living in Amman and those living in other governorates (71.4 ± 22.6 vs. 62.9 ± 22.2, *p* = 0.006). Similarly, higher QoL scores were reported by participants with a higher educational level (>12 years of education vs. ≤12 years), *p* = 0.003. Monthly income was also associated with overall QoL score, with those reporting an income > JOD 1000 per month also reporting a higher QoL, *p* = 0.016.

### 3.2. Unmet Supportive Care Needs

Needs as assessed with the modified SCNF-SF34 were greatest in the financial domain followed by the health system and information needs, physical and daily living and patient care and support domains, Table 2.

The top five unmet supportive care needs reported by survivors were: having a member of hospital staff with whom the survivor can discuss treatment and follow up issues with (48.1%; health system and information need), followed by money to cover living expenses (47.3%; financial need), to be informed about things one can do to help oneself get well (46.9%; Health system and information need), not sleeping well (46.3%; physical need) and help with work around the home (44.8%; physical need). The least prevalent needs reported were to be given information about sexual relationships (5.5%, sexuality need) and consultation for reproductive ability (6.7%, reproductive need); other needs are listed in Table 3.

### 3.3. Association between Supportive Care Needs and Selected Participants’ Characteristics

#### 3.3.1. Stage of Cancer

Late-stage diagnosis was associated with higher needs compared to early-stage diagnosis in the following domains: physical and daily living (44.6 ± 30.9 vs. 31.0 ± 28.6, *p* = 0.003), psychological needs (34.7 ± 25.4 vs. 23.4 ± 22.2, *p* = 0.002), patient care and support (39.6 ± 22.6 vs. 31.2 ± 22.8, *p* = 0.019), health system and information (44.2 ± 25.6 vs. 33.5 ± 26.5, *p* = 0.010) and financial needs (48.6 ± 39.7 vs. 33.1 ± 35.0, *p* = 0.007).

#### 3.3.2. Gender

Males reported more sexuality needs than females, (20.2 ± 28.6 vs. 10.9 ± 23.3, *p* = 0.009), in addition to higher patient care and support needs (38.6 ± 24.1 vs. 30.8 ± 23.0, *p* = 0.018).

#### 3.3.3. Comorbidities

Having comorbid chronic diseases was associated with relatively more physical needs, although the difference was not statistically significant (*p* = 0.07). However, cancer survivors with chronic diseases reported significantly less patient care and support (29.3 ± 22.9, *p* = 0.007), health system (30.8 ± 24.4, *p* = 0.002), reproductive (3.2 ± 16.7, *p* = 0.03) or financial needs (32.3 ± 34.5, *p* = 0.028). Psychological needs were also lower among survivors with comorbid conditions, but the difference did not approach statistical significance (*p* = 0.064).

#### 3.3.4. Marital Status

Marital status was significantly associated with physical needs (*p* = 0.003), and widows reported the highest physical needs (56.6 ± 31.2).

#### 3.3.5. Educational Level

Higher educational level among cancer survivors (more than 12 years of education vs. ≤12 years) was associated with less physical and financial needs (*p* = 0.004 and *p* < 0.001, respectively).

#### 3.3.6. Age

Reproductive need was higher among participants ≤45 years of age (*p* < 0.001).

However, psychological and sexual needs were not significant after Bonferroni correction.

#### 3.3.7. Place of Residency/Governorates

Cancer survivors residing in the capital city of Amman reported significantly fewer financial needs compared to participants residing in other governorates (32.8 ± 35.9 vs. 46.1 ± 37.6, *p* = 0.008). Similarly, sexuality needs were more prevalent among cancer survivors residing outside of Amman (19.4 ± 28.0 vs. 11.0 ± 23.5, *p* = 0.016).

#### 3.3.8. Monthly Income

Monthly income ≤ JOD 1000 was associated with significantly higher physical needs (36.1 ± 29.7 vs. 18.4 ± 23.2, *p* = 012) and financial needs (40.3 ± 36.9 vs. 14.4 ± 26.3, *p* = 0.004).

#### 3.3.9. Global Quality of Life Score

The overall quality of life (QoL) score was significantly and inversely associated with the mean need scores in the physical, psychological, health system and information and financial domains: *r* = −0.240, *p* < 0.001; *r* = −0.227, *p* < 0.001; *r* = −0.172, *p* = 0.008 and *r* = −0.335, *p* < 0.001, respectively, Table 4. 

### 3.4. Predictors of Unmet Supportive Care Needs

Using multivariate linear regression of mean scores, late-stage cancer diagnosis was independently associated with more physical (*p* = 0.032), psychological (*p* = 0.027), health system and information (*p* = 0.052), financial (*p* = 0.002) and overall need scores (*p* = 0.024). Cancer site was independently associated with more sexuality needs, and head and neck cancer survivors reported the highest needs (*p* = 0.029). Age above 60 years was independently associated with more physical needs (*p* = 0.008). Interestingly, the overall QoL score as reported by study participants emerged as a significant predictor of need on several domains: physical (*p* = 0.015), psychological (*p* < 0.0001), health system and information (*p* = 0.015), financial (*p* = 0.004) and the overall needs score (*p* = 0.0003). There was also a trend for association between the overall QoL score and sexuality needs (*p* = 0.067). Other associations that did not approach statistical significance are presented in Table 5 and Table 6. 

### 3.5. Qualitative Data from Cancer Survivors

Participants were asked open-ended questions at the end of the survey to identify their other needs and barriers to survivorship care. The top five unmet needs from a participant’s perspective that emerged following content and thematic analysis are illustrated in Table 7. Insurance coverage for other illnesses and treatment complications was the most frequently reported unmet need by survivors. Moreover, the need for follow-up on symptoms, treatment side effects and test results and the need for more information from the medical team, help to overcome financial problems and reducing waiting time were common themes reported by participants.

## 4. Discussion

In this population of cancer survivors treated at King Hussein Cancer Center, the overall needs as measured by the modified SCNS-SF were higher among patients diagnosed in late stages (stages 3 and 4), and there was a strong and independent association between the global health status/overall QoL score with overall needs. Late-stage diagnosis and QoL score were significant independent predictors of need in many domains, including physical, psychological, health system and information and financial needs. Needs assessed by the SCNS-SF were highest in the financial domain, which includes covering living expenses in addition to costs related to managing adverse effects associated with cancer treatment as well as costs of treating comorbidities, across all participants regardless of gender, age, cancer site, time since diagnosis or marital status. Health system and information, physical and daily living and patient care and support were also important needs reported by the study participants. The least prevalent needs reported by the study participants were information about sexual relationships and consultation for reproductive ability.

Mixed results have been reported in relation to supportive care needs and stage at diagnosis. For example, Sanders et al. reported no significant association between cancer stage at diagnosis and supportive care needs of lung cancer survivors [40], whereas a study of patients with resected melanoma reported those with more advanced stage had higher needs [41], similar to our findings. Our results are also compatible with some previous reports [42,43], while results reported from a recently published study on breast cancer revealed some differences [44], in which stage II, III and IV cancer patients had significantly lower needs than stage I patients. These discrepant findings may be explained by differences in study design (retrospective vs. prospective survey-based) and population (breast cancer vs. many cancers), resulting in variable outcomes. Though the population of cancer survivors in our study were all cancer-free and receiving no treatment except for hormonal maintenance at the time of survey, cancer stage at the time of diagnosis emerged as a significant predictor of unmet supportive care needs. A possible explanation may relate to high psychological needs among this group of survivors as reported by Hasegawa et al. [45] and supported by our results. This includes distress due to worry about the future, depressive feelings and fear of death. In addition, it could be related to the long and debilitating experience of aggressive treatment and the resulting long-term complications, which may be associated with more physical, health system/information and financial needs, similar to our findings.

The relationship between the QoL and supportive care needs of patients with cancer has recently been recognized [46,47], and a reduced QoL may be associated with psychological disorders or recurrence [48,49]. In our study, there was a strong, independent and inverse association between the global health status/overall QoL score with overall needs, and the QoL score was a significant predictor of need in many domains, including physical, psychological, health system and information and financial needs. This association highlights the importance of healthcare needs from the survivor’s point of view and provides evidence that a better quality of life could be associated with different degrees of patient needs; the higher the quality of life score, the lower the healthcare needs of cancer survivors. This is in line with previous research suggesting that unmet needs are more common in patients with worse quality of life [23,30,31]. The overall QoL score was significantly higher for participants with a higher educational level in our study. This is not surprising considering that a person’s educational level can influence their socioeconomic status and play a significant role in their understanding of their health needs and how they interact with the health system and make treatment decisions [50]. Furthermore, higher education and socioeconomic levels may also influence survivors’ ability to pay for supportive care services, overcome financial challenges and also gives them space to take time away from work without having to worry about their financial situation. In addition, higher QoL scores were reported by cancer survivors living in Amman, which may be explained by more support services available in the capital city and easier commuting and access to healthcare facilities.

The highest needs in our population were in the financial domain, although Jordanian cancer patients treated at KHCC are insured by the government for comprehensive cancer care [11]. However, after the completion of active treatment and transitioning to survivorship, governmental insurance does not cover any supportive care needs for cancer patients (e.g., psychosocial counseling, physiotherapy or nutritional counseling). Therefore, it was not surprising to find an association between monthly income and physical and financial needs. Other moderate to high unmet needs among our population were evident in the health system and information domain and physical/daily living domain. These included finding hospital staff to talk with, information on how to help oneself, help with sleeping and help with work around home, which supports previous findings by Alananzeh et al. [25]. Considering that around one-third of our population of participants was above the age of 60 years, and more than one-half reported other comorbidities; this may have increased their level of unmet needs, particularly physical needs, which was evident in the multivariate linear regression model.

Similar to other studies [25,51], our population of cancer survivors reported a low level of unmet sexuality needs. This may reflect a cultural taboo of not discussing sexual needs. Though it was expected to find more sexual needs in the younger group of cancer survivors, it was interesting to note the significant association between male gender and higher sexual needs. This was previously reported in the literature [24,52], where the sexual information needs of male cancer patients were frequently higher than those of female cancer patients. Other studies reported that female cancer patients had lower rates of consultation with hospital staff regarding sexual life than male cancer patients [53,54]. Similarly, other researchers found females to be passive in seeking sex-related information compared to men, and they might have little chance to communicate with their healthcare providers if the latter did not enquire about sexual issues with the patient [52]. This might be due to a lack of sex education, a lack of sexual autonomy, cultural factors or embarrassment with communication [55,56]. This requires in-depth research into how female cancer patients perceive their sexual needs in a variety of sociocultural contexts.

This study’s main strength is in the use of the validated SCNS-SF instrument for assessing needs among cancer survivors. The SCNS-SF was developed in an oncology population and encompassed multiple need domains. Nonetheless, we also asked participants to list problems and needs not included in the questionnaire in order not to miss uncovered areas of concern. While we examined survivorship needs in a convenience sample, there was representation of various cancer types and different Jordan governorates, and as a result, these findings have extended our understanding of unmet supportive care needs among Jordanian-Arab cancer survivors.

A limitation of the study was underrepresentation of males with around 70% of the study sample being females. This might be due to the fact that breast cancer is the most prevalent cancer in Jordan, with excellent outcomes and a large survivor population. However, more studies exploring gender-specific supportive care needs are required to better understand unmet needs among cancer survivors. Another limitation of the study was the use of self-report measures, which may have been subject to self-report bias. In addition, the study was conducted in a tertiary-level cancer center, where patients have greater access to healthcare providers including oncologists, nurse care coordinators and psychologists. Although this study can serve as a model to assess the needs of cancer survivors at a national level and extrapolated to other neighboring Arab countries considering the similarities in language and culture, it is difficult to generalize our findings to other countries and healthcare settings, and more research is needed in these countries to support or reject the findings in this study. Moreover, the level of needs among survey participants may have been lower than cancer survivors that did not participate, as this group of survivors may be more actively engaged in utilizing resources and services to address their needs, simply by virtue of responding positively to the survey. The cross-sectional nature of this study provides a limited dimension of time, making it difficult to determine causal relationships between the dependent and independent variables. However, in this study, variables such as date of diagnosis and date of birth were confirmed by the KHCC Cancer Registry. The impact of the COVID-19 pandemic on the healthcare needs among cancer survivors was not considered, therefore, we suggest performing studies in the future to overcome these limitations.

## 5. Conclusions

Late-stage cancer diagnosis and lower global health status/overall QoL score are associated with unmet supportive care needs among adult cancer survivors in Jordan. Overall, this needs assessment identified problem areas for targeting interventions across the Jordanian cancer survivor population, and understanding these findings suggests potential opportunities for intervention in addressing these gaps in care. Since, in our study, certain patient subgroups reported higher levels of needs and were more likely to experience problems and barriers related to their disease, caregivers and administrators should liaise with policy and decision makers to try to mend the gap and address unmet needs. Using QoL screening tools in survivorship clinics may help identify patients with unmet needs that require more attention from the physician and referral to supportive care services. Access to and use of supportive services should be promoted to the survivor cancer population provided financial coverage is secured to cover related costs. In this study, socio-demographic data such as gender, marital status, educational level, cancer type, cancer stage, comorbidities, time since diagnosis, age, monthly income, governorates as well as QoL were introduced as covariates in the regression analysis, the remaining variables were dropped out as no significant interaction was observed.

A future research direction by our group includes assessing the unmet supportive care needs in patients who survived a childhood cancer. Two studies are currently ongoing to assess needs among adult survivors of a childhood cancer and child survivors of a childhood cancer. These two groups of patients were considered unique and worthy of more focus in separate studies since their needs might be quite different from adult survivors of an adulthood cancer. Our goal is to comprehensively inform future research, survivorship programs and decision makers in Jordan and neighboring Arab countries of the supportive care needs of survivor cancer patients to be able to direct interventions and promote access relying on evidence-based data.

## Figures and Tables

**Table 1:** Characteristics of study participants, *n* = 240.

Characteristic	Level	*n* (%)
Gender	Male	73 (30.4)
	Female	167 (69.6)
Age	45 and less	58 (24.5)
	46–60	105 (44.3)
	More than 60	74 (31.2)
Income/month	≤1000	211 (91.7)
	>1000	19 (8.3)
Marital Status	Single	22 (9.2)
	Married	188 (78.3)
	Widow	20 (8.3)
	Divorced	10 (4.2)
Living condition	Alone	17 (7.1)
	With partner	184 (77)
	With family	38 (15.9)
Time Since Diagnosis	Five years and less	54 (23.0)
	6–10 years	94 (40.0)
	More than 10 years	87 (37.0)
Comorbidities	Yes	126 (52.5)
	No	114 (47.5)
Educational level	Less than high school	50 (20.8)
	Tawjihi (High school certificate)	59 (24.6)
	Diploma	50 (20.8)
	Bachelor degree	65 (27.1)
	Graduate studies	16 (6.7)
Educational level in years	≤12 years	109 (45.4)
	>12 years	131 (54.6)
Employment	None	147 (61.3)
	Full time	51 (21.3)
	Part time	21 (8.8)
	Others	21 (8.8)
Governorate	Amman	160 (66.7)
	Others	80 (33.3)
Type of Cancer	Breast	87 (36.3)
	Colorectal/Gastrointestinal	59 (24.6)
	Lymphoma/Leukemia/Hematological	72 (30.0)
	Head and neck	22 (9.2)
Stage at diagnosis	Early stage	168 (75.7)
	Late stage	54 (24.3)

*n* does not correspond to 240 in all variables due to missing values.

**Table 2:** Overall standardized scores by domain for the modified SCNS-SF (45 items).

Domain	Number of Items	Mean	Std. Deviation	Median
Physical and daily living	6	34.1	29.7	33.33
Psychological	12	26.1	23.5	22.92
Sexuality	3	13.8	25.4	0
Patient care and support	7	33.2	23.5	28.57
Health system and information	13	35.9	26.8	30.77
Financial	3	37.2	36.9	33.33
Reproductive	1	6.3	23.6	0
Overall needs	45	26.1	16.6	23.22

**Table 3:** Prevalence of Supportive Care Needs among study participants; all questions, 45 items (No Need vs. Some Need).

Domain	Supportive Care Need	No Need	Some Need
		*n* (%)	*n* (%)
Physical	Not sleeping well	129 (53.7)	111 (46.3)
Physical	Work around the home	132 (55.2)	107 (44.8)
Physical	Lack of energy and tiredness	136 (57.1)	102 (42.9)
Physical	Not being able to do the things you used to do	143 (59.6)	97 (40.4)
Physical	Feeling unwell a lot of the time	159 (66.5)	80 (33.5)
Physical	Pain	161 (67)	79 (33)
Psychological	Anxiety	133 (55.6)	106 (44.4)
Psychological	Fears about cancer returning	138 (57.7)	101 (42.3)
Psychological	Concerns about the ability of those close to you to cope with caring for you	147 (61.8)	91 (38.2)
Psychological	Uncertainty about the future	157 (65.7)	82 (34.3)
Psychological	Learning to feel in control of your situation	161 (67.4)	78 (32.6)
Psychological	Feeling down or depressed	164 (68.3)	76 (31.7)
Psychological	Feelings of sadness	168 (70)	72 (30)
Psychological	Feeling bored and/or useless	169 (70.4)	71 (29.6)
Psychological	Keeping a positive outlook	176 (73.9)	62 (26.1)
Psychological	Feelings about death and dying	177 (74.4)	61 (25.6)
Psychological	Concerns about the worries of those close to you	177 (74.7)	60 (25.3)
Psychological	Worry that the results of treatment are beyond your control	182 (76.8)	55 (23.2)
Sexual	Changes in sexual feelings	178 (74.8)	60 (25.2)
Sexual	Changes in your sexual relationships	185 (77.7)	53 (31.1)
Sexual	To be given information about sexual relationships	228 (94.5)	13 (5.5)
Patient Care	Reassurance by medical staff that the way you feel is normal	150 (63)	88 (37)
Patient Care	Hospital staff attending promptly to your physical needs	161 (67.4)	78 (32.6)
Patient Care	Waiting a long time for clinic appointments	161 (67.4)	78 (32.6)
Patient Care	Hospital staff acknowledging and showing sensitivity to your feelings and emotional needs	163 (68.2)	76 (31.8)
Patient Care	Waiting a long time to see your physician	162 (67.8)	74 (32.2)
Patient Care	More choice about which cancer specialists you see	186 (77.8)	53 (22.2)
Patient Care	More fully protected rights for privacy when you’re at the hospital	213 (89.1)	26 (10.9)
Health System	To have one member of hospital staff with whom you can talk to about all aspects of your condition, treatment and follow-up	124 (51.9)	115 (48.1)
Health System	To be informed about things you can do to help yourself to get well	127 (53.1)	112 (46.9)
Health System	To be informed about your test results as soon as feasible	133 (55.6)	106 (44.4)
Health System	To be informed about cancer which is under control or diminishing (that is, remission)	140 (58.8)	98 (41.2)
Health System	To be given explanations of those tests for which you would like explanations	144 (60.3)	95 (39.7)
Health System	To be given information (written, diagrams, drawings) about aspects of managing your illness and side-effects at home	145 (60.7)	94 (39.3)
Health System	To be given written information about the important aspects of your care	147 (61.5)	92 (38.5)
Health System	To be adequately informed about the benefits and side-effects of treatments before you choose to have them	149 (62.3)	90 (37.7)
Health System	The opportunity to talk to someone who understands and has been through a similar experience	153 (64)	86 (36)
Health System	To be treated like a person not just another case	168 (70.3)	71 (29.7)
Health System	To have access to professional counselling (e.g., psychologist, social worker, counsellor, nurse specialist) if you, family or friends need it	168 (70.6)	70 (29.4)
Health System	To be informed about support groups in your area	176 (73.6)	63 (26.4)
Health System	To be treated in a hospital or clinic that is as physically pleasant as possible	185 (77.4)	54 (22.6)
Financial	Money to cover living expenses	126 (52.7)	113 (47.3)
Financial	Money to treat other illnesses	138 (57.7)	101 (42.3)
Financial	Money to treat side effects of cancer and its treatment	151 (63.2)	88 (36.8)
Reproductive	Need consultation about the reproductive ability	222 (93.3)	16 (6.7)

**Table 4:** Univariate associations between selected participant characteristics and supportive care need mean scores.

Characteristic/Level	Physical	*p*	Psychological	*p*	Sexuality	*p*	Pt Care & Suppt	*p*	Health System & Info	*p*	Financial	*p*	Reproductive	*p*
Stage														
Early Stage	31.0	0.003	23.4	0.002	14.2	0.906	31.2	0.019	33.5	0.010	33.1	0.007	7.2	0.342
Late Stage	44.6		34.7		14.7		39.6		44.2		48.6		3.7	
Gender														
Male	30.2	0.178	23	0.182	20.2	0.009	38.6	0.018	38.6	0.291	39.3	0.574	2.8	0.129
Female	35.8		27.4		10.9		30.8		34.7		36.3		7.8	
Cancer Type														
Breast	35.7	0.939	24.6	0.775	12.5	0.255	33.1	0.985	36.4	0.773	34.8	0.547	5.2	0.681
CRC/GI	33.4		28.3		15.7		33.3		32.8		42.1		4.7	
Hematologic	32.9		25.2		11.1		32.8		36.9		38.2		9.2	
Head and Neck	33.3		28.2		22.7		35.1		38.6		30.7		5.7	
Comorbidities														
Yes	37.4	0.07	23.4	0.064	11.4	0.119	29.3	0.007	30.8	0.002	32.3	0.028	3.2	0.03
No	30.4		29.1		16.5		37.6		41.6		42.8		9.8	
Income/month														
≤1000	36.1	0.012	26.8	0.201	13.8	0.701	33.6	0.508	36.5	0.695	40.3	0.004	6.2	0.406
>1000	18.4		19.2		16.2		29.8		33.9		14.4		11.1	
Marital Status														
Single	23.8	0.003	25.4	0.762	7.2	0.006 *	33.8	0.389	38.5	0.497	35.2	0.964	11.4	0.361
Married	32.8		25.5		16.7		33.4		36.6		37.0		6.7	
Widow	56.6		31.7		0.0		26.7		27.5		40.0		0.0	
Divorced	36.7		27.1		0.0		42.5		33.3		40.8		0.0	
Educational Level														
≤12 years	40.2	0.004	27.3	0.472	13.7	0.968	32.5	0.645	27.7	0.976	36.6	<0.001	5.7	0.733
>12 years	29.1		25.1		13.9		33.9		26.0		35.0		6.8	
Age														
45 and less	32.2	0.792	32.3	0.027 **	18.4	0.032 **	34.1	0.666	41.5	0.213	40.8	0.664	21.1	<0.001
46–60	34.3		25.9		15.5		34.8		34.8		36.0		2.9	
More than 60	35.8		21.0		7.5		31.6		33.8		35.5		0.0	
Time Since Diagnosis														
≤5 years	32.5	0.881	26.7	0.963	13.5	0.651	30.5	0.322	39.6	0.172	39.6	0.789	8.0	0.692
6–10 years	35.1		26.2		15.6		36.1		38.1		37.9		5.4	
>10 years	34		25.6		12.1		32.2		31.9		35.3		6.6	
Governorates														
Amman	32.0	0.136	24.3	0.103	11.0	0.016	32.9	0.743	34.4	0.253	32.8	0.008	6.4	0.894
Others	38.1		29.6		19.4		34.0		38.7		46.1		6.0	
Global Health Status/QoL														
Mean QoL	34.1	<0.001	26.1	<0.001	16	0.226	33.2	0.404	35.9	0.008	37.2	<0.001	6.3	0.188

*p*, *p*-value; Pt Care & Suppt, Patient Care and Support; Health System & Info, Health System and Information; CRC/GI, Corolectal/Gastrointestinal. * Not significant after Bonferroni correction, the mean difference is significant at the 0.0083 level. ** Not significant after Bonferroni correction, the mean difference is significant at the 0.017 level.

**Table 5:** Multivariate linear regression of mean scores of Physical, Psychological, Sexuality and Patient Care and Support domain-specific needs on the modified SCNS-SF (45 items).

Characteristic/Level	Physical				Psychological				Sexuality				Patient Care & Support			
	MS	F Value	*p* Value	ηp^2^	MS	F Value	*p* Value	ηp^2^	MS	F Value	*p* Value	ηp^2^	MS	F Value	*p* Value	ηp^2^
Gender	168.44	0.22	0.6362	0.0017	439.21	0.98	0.3239	0.0072	980.00	1.26	0.2639	0.0105	725.66	1.70	0.1947	0.0122
Male																
Female																
Marital Status	2139.16	2.85	0.0396	0.0596	361.48	0.81	0.4921	0.0175	164.35	0.21	0.6466	0.0018	1063.59	2.49	0.0630	0.0517
Single																
Married																
Widow																
Divorced																
Educational Level	113.92	0.15	0.6973	0.0011	1606.89	3.59	0.0604	0.0257	296.88	0.38	0.5378	0.0032	1486.13	3.48	0.0643	0.0248
≤12 years																
>12 years																
Cancer Type	88.50	0.12	0.9494	0.0026	524.28	1.17	0.3235	0.0252	2417.90	3.11	0.0291	0.0727	87.31	0.20	0.8932	0.0045
Breast																
CRC/GI																
Hematologic																
Head and Neck																
Stage	3524.21	4.70	0.0319	0.0337	2251.44	5.03	0.0266	0.0356	138.92	0.18	0.6733	0.0015	793.94	1.86	0.1751	0.0134
Early Stage																
Late Stage																
Comorbidities	2503.91	3.34	0.0698	0.0241	859.85	1.92	0.1682	0.0139	1086.33	1.40	0.2396	0.0116	612.60	1.43	0.2332	0.0104
Yes																
No																
Time Since Diagnosis	804.58	1.07	0.3447	0.0157	6.46	0.01	0.9857	0.0002	152.04	0.20	0.8227	0.0033	399.77	0.94	0.3948	0.0135
≤5 years																
6–10 years																
>10 years																
Age	3731.92	4.98	0.0082	0.0687	995.80	2.22	0.1122	0.0317	1340.43	1.72	0.1828	0.0282	65.18	0.15	0.8587	0.0022
45 and less																
46–60																
More than 60																
Income/month	2472.94	3.30	0.0715	0.0239	77.93	0.17	0.6773	0.0013	178.44	0.23	0.6328	0.0019	433.36	1.01	0.3157	0.0073
≤1000																
>1000																
Governorates	469.77	0.63	0.4299	0.0046	586.40	1.31	0.2546	0.0095	2054.46	2.64	0.1067	0.0217	0.10	0.00	0.9879	0
Amman																
Others																
GHS/QoL	4573.97	6.10	0.0147	0.0432	7362.35	16.43	<0.0001	0.1078	2650.41	3.41	0.0674	0.0278	1067.82	2.50	0.1162	0.0179
Mean QoL																

GHS/QoL, Global Health Status/Quality of Life; ηp^2^ Partial Eta-Squared; MS, Mean Squares; CRC/GI, Colorectal/Gastrointestinal.

**Table 6:** Multivariate linear regression of mean scores of Health System and Information, Financial, Reproductive domains and overall specific needs on the modified SCNS-SF (45 items).

Characteristic/Level	Health System & Info				Financial				Reproductive				Overall Mean			
	MS	F Value	*p* Value	ηp^2^	MS	F Value	*p* Value	ηp^2^	MS	F Value	*p* Value	ηp^2^	MS	F Value	*p* Value	ηp^2^
Gender	373.81	0.60	0.4382	0.0044	631.27	0.56	0.4561	0.004	4486.40	7.21	0.0081	0.05	40.52	0.18	0.6745	0.0013
Male																
Female																
Marital Status	508.99	0.82	0.4833	0.0178	1100.32	0.97	0.4071	0.0207	483.44	0.78	0.5085	0.0167	454.34	1.99	0.1188	0.0414
Single																
Married																
Widow																
Divorced																
Educational Level	2015.06	3.26	0.0733	0.0234	2135.53	1.89	0.1715	0.0135	137.44	0.22	0.639	0.0016	61.48	0.27	0.6050	0.0019
≤12 years																
>12 years																
Cancer Type	160.14	0.26	0.8548	0.0057	500.60	0.44	0.7226	0.0095	1333.82	2.14	0.0974	0.0449	123.42	0.54	0.6559	0.0116
Breast																
CRC/GI																
Hematologic																
Head and Neck																
Stage	2377.36	3.84	0.0520	0.0275	10767.82	9.53	0.0024	0.0646	1291.26	2.08	0.1519	0.0149	1189.69	5.20	0.0241	0.0363
Early Stage																
Late Stage																
Comorbidities	252.12	0.41	0.5242	0.003	219.67	0.19	0.6600	0.0014	60.63	0.10	0.7553	0.0007	109.53	0.48	0.4901	0.0035
Yes																
No																
Time Since Diagnosis	428.90	0.69	0.5015	0.0101	163.68	0.14	0.8653	0.0021	817.32	1.31	0.2721	0.0188	55.37	0.24	0.7853	0.0035
≤5 years																
6–10 years																
>10 years																
Age	40.83	0.07	0.9361	0.001	895.58	0.79	0.4548	0.0114	1502.20	2.42	0.0931	0.0341	572.00	2.50	0.0857	0.035
45 and less																
46–60																
More than 60																
Income/month	212.96	0.34	0.5583	0.0025	2456.23	2.17	0.1427	0.0155	472.11	0.76	0.3851	0.0055	338.14	1.48	0.2261	0.0106
≤1000																
>1000																
Governorates	248.05	0.40	0.5276	0.0029	169.51	0.15	0.6991	0.0011	101.60	0.16	0.6867	0.0012	267.35	1.17	0.2815	0.0084
Amman																
Others																
GHS/QoL	3781.93	6.12	0.0146	0.043	9923.35	8.78	0.0036	0.0598	20.75	0.03	0.8553	0.0002	3526.58	15.42	0.0003	0.1005
Mean QoL																

GHS/QoL, Global Health Status/Quality of Life; ηp^2^ Partial Eta-Squared; MS, Mean Squares; Health System and Info, Health System and Information; CRC/GI, Colorectal/Gastrointestinal.

**Table 7:** Other needs identified by cancer survivors using open-ended questions.

Theme	Illustrative Quotes
Insurance	“The insurance doesn’t cover all clinics and this is why I have problems and private doctors refuses to treat other illness outside KHCC”
Follow-up	“I need the time interval between visits in the survival clinic to be 6 months instead of 1 year and doctors should ask for MRI every year to check for the disease recurrence and the endoscopy to be every 3 years instead of 5 years”
Communication	“I would like if the center can implement a way to deliver lab results by using a barcode for the patients to check it and I would like to join a group with patients of similar diagnosis to share the experience.”
Financial	“The bad financial situation affects the psychological and social status which affect the health, also there is a need to provide jobs for people who lost their previous jobs due to illness”
Service availability and waiting time	“KHCC should make satellite clinics for people who live far from hospital, this will make transportation easier and waiting time between clinics should be reduced”

## Data Availability

The data that support the findings of this study are available upon reasonable request.
